# The perceptual significance of high-frequency energy in the human voice

**DOI:** 10.3389/fpsyg.2014.00587

**Published:** 2014-06-16

**Authors:** Brian B. Monson, Eric J. Hunter, Andrew J. Lotto, Brad H. Story

**Affiliations:** ^1^Department of Pediatric Newborn Medicine, Brigham and Women’s Hospital, Harvard Medical SchoolBoston, MA, USA; ^2^National Center for Voice and Speech, University of UtahSalt Lake City, UT, USA; ^3^Department of Communicative Sciences and Disorders, Michigan State UniversityEast Lansing, MI, USA; ^4^Speech, Language, and Hearing Sciences, University of ArizonaTucson, AZ, USA

**Keywords:** speech perception, acoustics, singing, voice, high-frequency

## Abstract

While human vocalizations generate acoustical energy at frequencies up to (and beyond) 20 kHz, the energy at frequencies above about 5 kHz has traditionally been neglected in speech perception research. The intent of this paper is to review (1) the historical reasons for this research trend and (2) the work that continues to elucidate the perceptual significance of high-frequency energy (HFE) in speech and singing. The historical and physical factors reveal that, while HFE was believed to be unnecessary and/or impractical for applications of interest, it was never shown to be perceptually insignificant. Rather, the main causes for focus on low-frequency energy appear to be because the low-frequency portion of the speech spectrum was seen to be sufficient (from a perceptual standpoint), or the difficulty of HFE research was too great to be justifiable (from a technological standpoint). The advancement of technology continues to overcome concerns stemming from the latter reason. Likewise, advances in our understanding of the perceptual effects of HFE now cast doubt on the first cause. Emerging evidence indicates that HFE plays a more significant role than previously believed, and should thus be considered in speech and voice perception research, especially in research involving children and the hearing impaired.

## INTRODUCTION

In 1907 and 1908, the following acoustical observations were reported in *Philosophical Magazine* by physicist Lord Rayleigh:

“The other branch of the subject, which I had hoped to treat in this paper, is the discrimination between the front and back position when a sound is observed in the open; but various obstacles have intervened to cause delay. Among these is the fact that (at 64 years of age) my own hearing has deteriorated. … Now, as I find to my surprise, I fail to discriminate, even in the case of human speech. It is to be presumed that this failure is connected with obtuseness to sounds of high pitch, such as occur especially in the sibilants. … If, as seems the only possible explanation, the discrimination of front and back depends upon an alteration of *quality* due to the external ears, it was to be expected that it would be concerned with the higher elements of the sound.”

(Rayleigh, 1907, pp. 230–231)

“…Mr. Enock [Rayleigh’s assistant] is able in many cases to discriminate front and back when the voice is used normally. But I find that both indoors and outdoors he could be deceived. Thus when standing on the lawn only a short distance in front of him, but facing *from* him, I gave the numerals, he judged that I was behind him, and this erroneous judgment was not disturbed even when I conversed freely with him. … Probably the turning away of the speaker softens the sibilants and other high elements in the sound. … The repetition and extension of these observations would be of interest.”

(Rayleigh, 1908, pp. 240–241)

Rayleigh’s commentaries on these phenomena are among the earliest published records we have on the perceptual effect of high-frequency energy (HFE) in speech and voice. In the century since Rayleigh’s observations, however, study of the acoustical energy in human speech and voice communication has typically been restricted to the frequency range below ~5 kHz. Consequently, while the human audible frequency range extends up to about 15 kHz for most individuals and to 20 kHz for younger adults and children, the term “high frequency” in the speech and voice literature often refers to frequencies anywhere from 2 to 5 kHz (e.g., Hornsby and Ricketts, 2003), or, more rarely, up to 6 or 8 kHz (e.g., Baer et al., 2002). This low-frequency research trend has resulted in a good understanding of the phonetic value of the lower formants of speech and the fundamental frequency of speech. It has also resulted, however, in a paucity of information about HFE (defined here as the energy in the 8- and 16-kHz octave bands, or 5.7–22 kHz) in human speech and vocal communication, including what energy and information exists in this region, how it is produced and/or modified, and how it is perceived by a human listener.

Based on various recent findings, it is tempting to speculate how HFE research could augment our current understanding of speech and singing. For example, the most common complaint made by users of hearing aids (which are only now beginning to amplify frequencies in this range) continues to be communication difficulty in noisy environments (Kochkin, 2010). Could it be that this problem would be partially ameliorated by restoring the listener’s ability to receive a high-fidelity full-bandwidth signal? Evidence suggests that degradation of HFE hearing in otherwise normal listeners can give rise to difficulties perceiving speech in noise (Badri et al., 2011). Corroborating this idea is the finding that some cortical neurons shown to respond specifically during competing speech tasks are tuned to frequencies above 6 kHz (Mesgarani and Chang, 2012). Perhaps part of the solution to the infamous “cocktail party problem” is embedded in HFE. More drastically, perhaps speech perception experiments are inherently confounded without the use of full-bandwidth high-fidelity stimuli because this practice deprives listeners of useful information available in the high frequencies. This confound might be especially significant in studies involving children, as data show that child word-learning rates decrease dramatically with deprivation of HFE (Pittman, 2008). The review that follows also sparks speculation regarding the importance of high-frequency hearing for tasks such as accurate diagnosis of speech disorders by a clinician, judgment of speech/voice quality by a clinician or singing voice teacher, and establishing the gender, identity, and emotion of a talker.

Various justifiable reasons have existed for the focus on low-frequency energy (and consequent neglect of HFE) in speech perception research. Some of these reasons date back to the early- and mid-twentieth century when much of the foundational work for current speech science research was laid. As is sometimes the case, a historical precedent can constrain research and theory long after the rationale for the precedent has vanished. In this review, we will describe the reasons for the neglect of HFE and why it seems time to revisit the upper portion of the spectrum in research on speech and song perception. After a discussion of the historical perspective, we will present a review of the acoustical analysis and the perceptual relevance of speech and voice HFE.

### HISTORY OF SPEECH RESEARCH

Tracing through the history of modern speech science reveals an upsurge of interest in the field that appears to have occurred around the turn of the 20th century, concomitant with the advent of the telephone. Bell Laboratories, the research branch of the American Telephone and Telegraph Company (AT&T), produced many of the well-known speech researchers and seminal papers in speech science. The following perspective on speech research at that time was written by Harvey Fletcher, who for many years worked and directed research at Bell Labs:

“It is evident that progress in the knowledge of speech and hearing has a great human interest. It will greatly aid the linguists, the actors, and the medical specialists. It may lead to improved devices which will alleviate the handicaps of deaf and dumb persons. Furthermore this knowledge will be of great importance to the telephone engineer, and since the telephone is so universally used, any improvement in its quality will be for the public good.”

“These humanitarian and utilitarian motives as well as the pure scientific interest have already attracted a number of scientists to this field. Now that new and powerful tools are available, it is expected that in the near future more will be led to pursue research along these lines.”

(Fletcher, 1922)

Telephone technology brought a myriad of research pathways for scientists to pursue, and, true to Fletcher’s prophecy, many were led to do so. The great interest lay in improving the efficiency of the telephone, without sacrificing *articulation* (i.e., intelligibility). Such improvements entailed determining which aspects of speech were sufficient and necessary for intelligible conversation. This led Fletcher and others at Bell Labs to study the acoustical energy distribution of speech (Crandall and MacKenzie, 1922) and the frequency-dependence of speech intelligibility by means of so-called “articulation tests” (Fletcher and Steinberg, 1930). Articulation tests played a major role in the development of telephony and received continued interest during the subsequent world war, as focus was shifted to the need for intelligible communication in drastically poor acoustical environments associated with warfare (see Fletcher and Galt, 1950, p. 90).

The work resulted in a theoretical model known as the *articulation index* (French and Steinberg, 1947; Fletcher and Galt, 1950), which greatly influenced subsequent speech research. The model predicted the loss of articulation (intelligibility) of a speech communication event given the separate degrading factors of the communication system and environment. Results of the articulation tests indicated about 95% accuracy for speech low-pass filtered at 4 kHz. The articulation index achieved its maximal value of 1 with a cutoff frequency of 7 kHz, suggesting that HFE was unnecessary for intelligible speech. These results are in part why the telephone bandwidth was restricted to a small portion of the full audio bandwidth (300–3.4 kHz)—a specification that persists in mobile phone technology today.

Other technological difficulties prohibited study of HFE during these early years. For example, audio instrumentation at the time was unable to represent HFE in speech with high-fidelity. “Beyond 5,000 cycles per second, the energy is so low as to be impossible of measurement with the apparatus used” (Crandall and MacKenzie, 1922). This led one researcher to emphatically declare, “…the *rustle* of a paper has never been heard over the microphone!” (McLachlan, 1931). By the time improvement was seen in the frequency response of transmission systems, it seems that HFE in voice and speech had already been relegated to the back shelf of scientific research. Phrases found in the literature are telling: “…throughout the speech range (600–4,000 cycles)…” (Fletcher and Wegel, 1922); “A few readings have also been made from 4600 to 10,900 cycles, but the amount of power contained within this range is usually very small” (Wolf et al., 1935); “…very little energy was found above 4200 cycles…[indicating] that it was unnecessary to carry the analysis to higher components” (Stout, 1938); “the range of frequencies important for the transmission of speech, i.e., from about 250 to 7000 c.p.s” (Stevens et al., 1947); “high speech frequencies…,” referring to the frequencies above 2375 cycles per second (Pollack, 1948).

Continued development of signal processing technology—most notably the move toward digital signal processing (DSP)—greatly contributed to speech and voice analysis techniques generally. Yet HFE remained an obscurity, with these advancements doing little to draw scientists toward the higher frequencies of the speech signal (e.g., Klatt, 1980; Klatt and Klatt, 1990). Early on, it is possible that this was due to limited processing speeds and sampling rates associated with DSP, which consequently limited the frequency bandwidth of analysis. When these features were improved, however, few exploited them in speech research, leading to a prevailing trend not uncommon in speech research even today (i.e., the continuing use of sampling rates of 8, 16, 22.1, or 24 kHz, when 44.1- and 48-kHz sampling rates are readily available).

### ACOUSTICAL CHARACTERISTICS OF HFE

While the history of speech research (i.e., the articulation index and limitation of technology at hand) provides reasons why HFE and its effects in speech were disregarded, several other acoustical factors may have also played a role. For example, an inspection of the typical speech spectrum reveals that acoustical energy at frequencies above 5 kHz tends to be greatly decreased in energy level (from 20 to 40 dB down) compared to the low-frequency portion of the spectrum (Moore et al., 2008; Monson et al., 2012a). With this rapid fall-off, one might question whether differences in this portion of the spectrum are even audible.

Added to this fact is an array of new difficulties that arise when attempting to examine HFE, which must be considered carefully when setting up experimental conditions. For example, research on HFE requires higher quality transducers and recording/playback equipment to maintain an accurate acoustical representation of the speech or voice signal. While great improvements have been made in the frequency response characteristics of microphones and loudspeakers today, not all are manufactured to have a flat response out to 20 kHz – a necessity for true high-fidelity reproduction of audio signals, and particularly HFE. Greater care is also required in matters of microphone and loudspeaker placement due to the directionality and scattering effects commonly found with high-frequency sound propagation. This in turn requires an understanding of the acoustical characteristics of the test environment, of possible reflective surfaces, etc.

High-frequency energy generation and propagation through the vocal tract becomes increasingly difficult to model due to the decrease in the acoustic wavelength as frequency increases. Higher frequencies with smaller wavelengths generate higher-order acoustic modes that violate the planar propagation assumption under which most propagation models operate. While non-planar propagation has been investigated in such applications as aircraft noise propagation through a duct (Schultz et al., 2006), the complexity of a high-frequency mathematical model for voice and speech is increased due to the irregular shape of the vocal tract and the dynamic nature of the vocal tract shape during vocal production. Despite these issues, some efforts in this arena have been made using three-dimensional vocal tract models (Takemoto et al., 2010).

**FIGURE 1:**
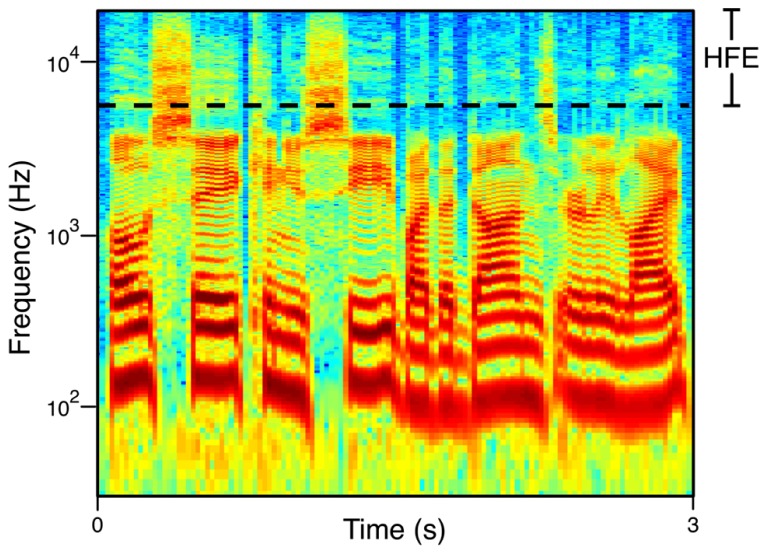
**Broadband spectrogram of speech.** The phrase shown is “Oh say can you see by the dawn’s early light” spoken by a male talker. The HFE range (the 8- and 16-kHz octaves) is demarcated.

Finally, while the HFE band (5.7–22 kHz) seems to take up a large portion of the audio bandwidth on a linear scale, the tonotopic organization of the ear is characterized as being roughly logarithmic in nature. As frequency increases, critical bandwidths increase (Zwicker, 1961), and frequency discrimination decreases (Moore, 1973). In essence this means that the HFE band is currently understood to be a rather small percentage of the physiological and perceptual audio bandwidth (**Figure 1**).

### MOTIVATION TO STUDY HFE

The combination of historical and physical factors reviewed here appears to have cast HFE out of the limelight of speech research for the majority of the 20th century. However, what should be noted from the above discussion is that, while the speech band above 5 kHz has been shown to be *unnecessary* and/or *impractical* for many applications of interest, the higher portion of the spectrum has not been shown to be perceptually *insignificant*. There is now an accruing interest in applications utilizing HFE in speech and voice. For example, recording studio engineers and live sound engineers regularly manipulate this “treble” frequency range for singing and speaking voices using equalization techniques. These techniques tend to be based on the perception of the engineer. Until recently the actual sensitivity of the engineer, or the audience of listeners, to changes in HFE in voice was unknown. Further, while standard telephony has been restricted to the frequency range below 4 kHz, so-called “wideband” telephony or “HD-voice” is now being integrated in applications within digital communication and Internet protocol (Geiser, 2012; Pulakka et al., 2012). This technology expands the bandwidth of transmission to at least 7 kHz. The perceptual effect of this bandwidth expansion has not been studied in detail. As a final example, some researchers have claimed the need for extending the bandwidth of augmentative hearing devices for communication purposes (e.g., Stelmachowicz et al., 2001) and some efforts are being made in this direction (Keidser et al., 2011; Moore et al., 2011). The usefulness of such bandwidth extension, and what frequency range is actually necessary, is still debated (reviewed in Moore et al., 2008).

## ACOUSTICAL ANALYSIS OF HFE

### SPECTRAL CHARACTERISTICS

Some studies exist that involve acoustical analysis of HFE in speech and voice. A handful of these have reported energy levels in the long-term averaged spectrum (LTAS) of speech, beginning with Sivian (1929). He reported that relative levels for two HFE bands (5.6–8 and 8–12 kHz) were some 50 dB down from maximum levels obtained in lower-frequency bands. While the HFE levels he reported were likely distorted by the equipment available to him, he made the following interesting observations regarding HFE: average relative HFE levels for female voices (*n* = 3) were 4–8 dB higher than levels for male voices (*n* = 5); and relative HFE levels were affected neither by recording distance (2 inch vs. 3 ft), nor by vocal intensity (“normal-conversational” vs. “high-declamatory”).

Dunn and White (1940) reported data for the same two HFE bands (5.6–8 and 8–12 kHz). Similarly, their results showed that female HFE levels (*n* = 5) were higher than male levels (*n* = 6), but both bands were 30–45 dB down in relative level. Byrne et al. (1994) performed an international study that showed how 1/3-octave band LTAS levels differed across languages, countries, and dialects. Included in their report were levels for 1/3-octave bands with center frequencies of 6.3, 8, 10, 12.5, and 16 kHz. In general, women had higher levels of HFE than men across languages, averaging differences of 2–3 dB for HFE bands (the notable exceptions being Vietnamese, Russian, and Singhalese, where HFE levels were fairly comparable for the two genders). Their data also showed that English speakers from Australia and New Zealand exhibited higher levels of HFE than those from the United States, or United Kingdom. Interestingly, even within the United States talkers from Memphis, TN had higher HFE levels than those from Columbus, OH, suggesting that HFE level might be influenced by dialect. A considerably different result from previous studies was that HFE bands were only 18–22 dB down from the bands with maximum energy.

A more recent LTAS study by Moore et al. (2008) reviewed the two studies mentioned in the previous paragraph and included a comparison to their own measurements of 1/3-octave band levels. The gender differences were consistent with previous results (*n* = 17, 8 female), but HFE levels varied greatly across frequency band, from 16 dB down at 6.3 kHz to 28 dB down at 16 kHz. The talkers were all British in this study, however, and comparison with the results from only the United Kingdom in Byrne et al. (1994) reveals somewhat similar values.

Monson et al. (2012a) performed an in-depth study specifically targeting HFE sound pressure level (SPL) differences in the LTAS calculated for several different conditions (*n* = 15, 8 female). Significant level differences were found between genders and between production levels (soft, normal, loud). Interestingly, significant gender differences were found only for the 16-kHz octave (higher levels for females), while the 8-kHz octave level showed no significant difference. HFE level increased significantly for both speech and singing as production level increased (soft to normal to loud), with loud speech exhibiting the highest HFE levels (see also Shadle and Mair, 1996). Mean HFE level (the combined 8- and 16-kHz octave levels) in normal speech was 47 dB SPL, 15 dB down from the overall mean level of 62 dB SPL. This level was higher than that found by Moore et al. (2008), whose data show HFE level to be about 45 dB SPL associated with an overall mean speech level of 65 dB SPL. **Table 1** summarizes the HFE levels reported in these studies.

**Table 1:** High-frequency energy (HFE) levels reported from five separate studies when the overall level is scaled to be 65 dB SPL.

Study	HFE Band	HFE Level	
		Male	Female
Sivian (1929)	5.6–12 kHz	~13 dB	~21 dB
Dunn and White (1940)	5.6–12 kHz	~24 dB	~30 dB
Byrne et al. (1994)	5.7–18 kHz	43.7 dB	46.3 dB
Moore et al. (2008)	5.7–18 kHz	45.2 dB	
Monson et al. (2012a)	5.7–22 kHz	48.2 dB	51 dB

*
Levels are shown separately for male and female subjects where available.*

It is important to keep in mind the recording methods used in these separate studies. Dunn and White (1940) recorded with a microphone located 30 cm *directly in front of* the mouth. Byrne et al. (1994) made recordings with the microphone located 20 cm in front of the mouth, on the same horizontal (transverse) plane as the mouth, but *at a 45°* angle in the azimuth (laterally). Moore et al. (2008), on the other hand, recorded at a distance of 30 cm directly on-axis from the mouth, but 15 cm *below* the mouth, while Monson et al. (2012a) recorded from 60 cm directly on-axis from the mouth *at the same height* as the mouth. While the recording distance may not have had a significant influence on relative HFE levels (Sivian, 1929), Monson et al. (2012b) showed that the directional nature of high-frequency radiation from the mouth significantly affects HFE level at different recording angles, particularly beyond 30°. This may account for some of the discrepancies found between these studies and highlights the importance of using a recording angle of no more than 30° when recording for high-fidelity purposes. Moreover, recording distance and angle (both horizontal and vertical) should always be reported for any research where speech recordings are made or used.

### FRICATIVES

Most of the speech and voice studies that have included HFE in acoustical analysis have been focused on the production of consonants that show significant spectral energy at frequencies between 5 and 10 kHz (i.e., fricatives). These efforts have typically been in search of acoustical parameters that successfully distinguish fricative classes. While an exhaustive review of the entire literature on fricative noise is not given here (for such a review, see Maniwa et al., 2009), a few studies are noteworthy with regard to the current review.

One of the seminal papers on this topic was that of Hughes and Halle (1956) in which they presented analysis of both voiced and voiceless fricatives spoken by two male speakers and one female speaker. Surprisingly, their work is often cited for its illustration of the variability of fricative production across subjects, despite the small population used. No mention is made of where or how the recording microphone was set up, but the figures they present show data out to 10 kHz. They report that “the three classes of fricatives (labial, dental, and palatal) are quite consistent,” particularly within subject. Their data show that spectral peaks for /s/ and /z/ (between 3 and 8 kHz) are consistently higher in frequency than peaks for /ʃ/ and /ʒ/ (between 1 and 3 kHz), and peaks for /f/ and /v/ (around 8–9 kHz) are fairly consistently higher in frequency than peaks for /s/ and /z/. (While the authors do not make the latter observation regarding /f/ and /v/, it is evident from their data.)

Shadle and Scully (1995) showed that spectral peaks of the fricative /s/ can be context-dependent. They demonstrated the effect of lip rounding during the production of /s/. The lip rounding caused the /s/ spectral peak to shift from around 5.5 kHz in the words /pasa/ and /pisi/ to 7.5 kHz in the word /pusu/ within the same subject. These data are pertinent because the major spectral differences found in differing contexts were above 5 kHz, suggesting the potential for HFE to be manipulated volitionally through vocal tract modification (e.g., lip rounding). While vocal tract resonances are known to shift during lip rounding, the effect on the high-frequency peak in this study was the opposite of what was observed for lower formant peaks, which shifted slightly lower in frequency. Although it is generally accepted that vocal tract resonances in the frequency range below 5 kHz shift downward with lip rounding (Fant, 1960), the apparent upward shift of the high-frequency peak in this study is likely due to a change in source properties from turbulence noise (alone) to turbulence noise plus a whistle.

Jongman et al. (2000) examined several potential cues for place of articulation of English fricatives. They recorded 20 talkers at a 45° lateral angle from the mouth at 15 cm speaking the eight English fricatives in consonant-vowel-consonant (CVC) syllables using six vowels /i,e,æ,*a*,o,u/. They reported that voiceless phonemes /f/ and /θ/ had higher-frequency spectral peak locations (about 8 kHz) than their voiced counterparts /v/ (7.5 kHz) and /ð/ (7 kHz), as well as /s,z/ (7 kHz), which in turn had higher frequency locations than /ʃ,ʒ/ (4 kHz). They also reported a main effect of gender, showing that women had higher mean spectral peak locations than men for /θ,ð/ (7.8 vs. 7.2 kHz), /s,z/ (7.5 vs. 6.2 kHz), and /ʃ,ʒ/ (4.3 vs. 3.3 kHz), but not for /f,v/ (7.6 vs. 7.8 kHz). Spectral peak location was found to significantly distinguish place of articulation. Maniwa et al. (2009) confirmed the latter result (though some values were slightly different), and extended the number of acoustic parameters used to classify fricatives. Similarly, Monson et al. (2012a) demonstrated that voiceless fricatives exhibit HFE octave and third-octave level differences that distinguish phonemes. Notably, the only significant differences found between /f/ and /θ/ were in the 8- and 10-kHz 1/3 octave bands.

Other efforts in this area have included simulation of fricatives (e.g., Heinz and Stevens, 1961) and children’s production of fricatives (e.g., Pentz et al., 1979). These topics are not reviewed further here, but the reader is referred to these papers for more detail.

### VOICE/SINGING VOICE

Fry and Manen (1957) performed one of the earliest notable studies on HFE. Their work is unique in two respects. First, whereas most of the early studies previously mentioned included HFE only as part of a data plot or data report (giving little to no commentary), these researchers analyzed and reported data specific to HFE in voice. Second, their study consisted of acoustical analysis of the singing voice. This study reported on spectral changes that occurred when singing in the three different moods of “aggressive,” “joyful,” and “fearful.” They found that articulatory changes for these moods “seem to influence mainly the components in the higher frequency range from about 4000 to 8000.” Specifically, they showed that a baritone singing the vowel /i/ at 250 Hz had strong harmonic components up to 8 kHz in the “aggressive” mood, up to 6 kHz in the “joyful” mood, and no measurable components above 4 kHz in the “fearful” mood. No mention is made of how the singer was recorded, but the fact that the harmonics were noticeably altered in samples of isolated voice is intriguing given that most of the work in HFE to date has been on the noise of fricative consonants. These results prompt questions regarding the role of HFE in the perception of a singer or talker’s mood.

Work by Shoji et al. (1991) represented the first attempt at some detailed characterization of HFE in isolated voice (i.e., without consonants). They reported mainly on spectral peaks found above 5 kHz. Their major findings appear to be the presence of a spectral dip around 5 kHz for all subjects tested—found later to be caused by an antiresonance associated with the piriform fossa (Dang and Honda, 1997)—and higher relative levels of spectral peaks in the HFE range for the vowels /a/, /e/, and /i/ compared to levels for /o/ and /u/. They also mention that gender had a significant effect on both frequency and level of HFE spectral peaks (higher for females in both cases).

Shoji et al. (1992) followed up on their own work by introducing an acoustic measure called the “high-frequency power ratio” to be used by clinicians to distinguish normal from breathy voices. This measure is the ratio of acoustical power above a given frequency (*f*_c_) to the total acoustical power. Using 16 normal (7 female) and 24 breathy voice (14 female) individuals phonating on the vowel /*a*/, they compared high-frequency power ratios calculated with *f*_c_ values ranging from 1 to 10 kHz. They found that using *f*_c_ = 6 kHz significantly separated normal voices from breathy voices and suggested that if the measured ratio (using this frequency) is greater than -30 dB the voice can be considered breathy; that is, above 6 kHz breathy voice has a higher amplitude than normal voice, on average. Similarly, Valencia et al. (1994) reported significant effects on energy levels of two HFE bands (6–10 and 10–16 kHz) in averaged spectra of normal vs. dysphonic voices. In this case, 12 female voices were analyzed (six normal, six vocal nodule patients), resulting in significantly higher levels (both absolute and relative) in both HFE bands for the dysphonic group.

Titze and Sung (2003) found a fairly consistent peak located near 10 kHz in the singing voice spectra of 16 trained tenors. They suggest that this is a second “singer’s formant” brought about by the second resonance of the epilaryngeal tube, whose first resonance (around 3 kHz) is attributed as the source of the well-known “singer’s formant cluster” found in spectra of trained Western classical singers. They also suggested that this second singer’s formant could reach perceptual significance in the right acoustical environment.

Ternström (2008) described some general characteristics of HFE in singing voice, including a report of harmonic energy up to 20 kHz in the singing voice spectrum. Ternström (2008) further reported that this energy was also present in female singing on the nasal /n/! (Nasals are typically characterized in speech as having a strong fundamental frequency with very little energy in the higher harmonics). Clearly there is still much to be understood regarding this high-frequency range. It is possible that singing voice research is an appropriate medium through which one can explore the questions of interest. This idea is strengthened by the finding that singing voices exhibit significantly higher HFE levels than speech for soft and normal conditions (though not for loud conditions; Monson et al., 2012a).

### GENERATION MECHANISM

As a final note regarding acoustical analysis of HFE, there are some efforts being made to characterize the generation mechanism for at least the broadband component of speech HFE. Isshiki et al. (1978) showed spectrogram data up to 8 kHz for turbulent flow noise generated by a physical model of the vocal folds with a gap in the posterior glottis. More recently, Zhang et al. (2004) gave experimental results of broadband flow noise generated at the glottis with a physical model. They showed spectral data out to 13 kHz for flow through normal, convergent, and divergent glottal orifices. Narayanan and Alwan (2000) attempted computational modeling (out to 10 kHz) of flow noise for fricatives using magnetic-resonance imaging (MRI) data, comparing their results to actual fricatives produced by human subjects. These efforts will likely continue if HFE is found to play a significant perceptual role in speech and voice.

## PERCEPTUAL RELEVANCE OF HFE

As shown here, relatively little detailed characterization of speech and voice HFE has been documented to date. That which has been documented is largely limited to the frequency range between 5 and 10 kHz for a subset of consonants. Very little characterization of speech or voice HFE above 10 kHz is available (although see, for example, Tabain, 1998).

Is such characterization necessary? Evidence suggests that human listeners can utilize HFE in speech. Snow (1931) showed that listeners discriminated normal speech from low-pass filtered speech with 60% accuracy (just slightly above chance) at cut-off frequencies of 8.5 kHz for male speech and 10 kHz for female speech. Listeners achieved 80% accuracy when cut-off frequencies were lowered to 7 and 9 kHz for male and female speech, respectively. While very little commentary was given on the perceptual effect of HFE on speech, Snow did write, “A frequency range of 100–10,000 cycles was shown to be entirely satisfactory for speech.” (The apparatus used in this study had a practical upper-frequency limitation of ~15 kHz.)

Monson et al. (2011) found that HFE level differences were detectable for normal-hearing listeners (*n* = 30). Difference limen (DL) estimates were obtained by incrementally increasing and decreasing the separate 8- and 16-kHz octave bands in recordings of isolated sung vowel sounds. Median DLs in the 8-kHz octave were 5–6 dB for loud singing stimuli. Minimum DLs ranged from 1 to 5 dB in both the 8- and 16-kHz octaves. When running speech and singing were used as stimuli, listeners showed even greater sensitivity to HFE level changes (Monson et al., 2014).

A follow-up hypothesis from these studies is that if humans can detect level changes (or absence vs. presence) of HFE in speech and voice, then HFE may contain information relevant to the percepts of speech and voice. As the following review will indicate, the perceptual studies on HFE to date have revealed several of the percepts of voice and speech in which HFE has a potential role.

### QUALITY

Most people would likely not be surprised to hear that HFE, or “treble,” plays a role in perception of music (see Moore, 2012), in which quality is typically a main objective. It is not obvious, however, whether this role would transfer to speech, in which transmission of a message is typically the main objective. Olson (1947) conducted a perceptual preference experiment using an acoustical filter (a wall with panels that could be rotated open or closed) to low-pass filter orchestral music at 4 kHz. He found that the majority of listeners preferred the full-range music. He remarked that listeners preferred full-range speech to filtered speech as well, though he showed no data on this. He did report, however, that listeners described the low-pass filtered speech with terms primarily indicating a change in quality (e.g., “muffled,” “muddy,” “mushy,” “lacking in intimacy,” “pushed back,” and “not as intelligible”). This study was one of the first to indicate the importance of HFE on qualitative percepts of speech. Monson et al. (2011, 2014) confirmed this finding in reports of listener responses describing human voice HFE level changes in mostly qualitative terms very similar to those given by Olson (1947).

More recent results from Moore and Tan (2003) demonstrate that the percept of “naturalness” is affected by HFE. Part of their study examined “naturalness” scores for band-pass filtered speech and music. The speech stimulus consisted of the concatenation of two sentences, one spoken by a man and the other by a woman. Stimuli were presented over headphones to 10 normal-hearing listeners. Listeners were asked to rate the randomly presented stimuli on a scale of 1–10 where “10” represented “very natural – uncolored” and “1” represented “very unnatural – highly colored.” With the lower cut-off frequency (*f*_l_) set at 55 Hz, changing the upper cut-off frequency (*f*_u_) from 16.9 to 10.9 kHz had little effect on the mean perceived naturalness score for the speech stimulus. However, a change in *f*_u_ from 10.9 to 7 kHz markedly decreased the mean naturalness score from nearly 8 to ~5.5. Another large decrease was seen in the step to 5.6 kHz (mean score about 3.5), with smaller successive decreases from there. A very similar effect was seen with *f*_l_ set at 123 Hz, except that the first *f*_u_ step (16.9–10.9 kHz) decreased the mean score from a little over 9 down to 8. However, this appears to have occurred because the 123 Hz–16.9 kHz condition received a higher mean naturalness score (9+) than the 55 Hz–16.9 kHz condition (about 8), which is a bit curious. The authors offer no explanation for this phenomenon.

It is notable that the largest decrease in naturalness score due to a change in *f*_u_ occurred for the step from 10.9 to 7 kHz in both *f*_l_ conditions (2.5 point decrease), and the second largest decrease (2 point decrease) occurred for the step from 7 to 5.6 kHz in both *f*_l_ conditions, suggesting that HFE plays a significant role in the percept of naturalness of speech. It is also worth noting that this pattern was *not* seen for the music stimulus (consisting of a jazz combo of piano, bass, and drums). Rather, decreasing *f*_u_ for music caused more consistently sized decreases in naturalness score (i.e., with *f*_l_ = 55 Hz, scores for *f*_u_ = 10.9, 7, and 5.6 kHz were about 8, 6.5, and 5.5, respectively; similar decreases were seen with *f*_l_ = 123 Hz).

Ternström and Howard (2004) suggested that a percept of “buzziness” in synthesized singing was affected by HFE. In their study the spectral level of the 6–8 kHz band in one real and one synthesized baritone singing stimulus was varied in 6-dB steps from -12 to +12 dB relative to the original level. They reported that each 6-dB increase caused a significant increase in perceived “buzziness” by 25 listeners. Füllgrabe et al. (2010) found that normal-hearing listeners (*n* = 4) significantly preferred both the “pleasantness” and “clarity” of speech low-pass filtered at 10 kHz to that filtered at 7.5 kHz, which in turn was significantly preferred to speech filtered at 5 kHz. This was true for both male and female speech.

### DISORDERED SPEECH

Clinically, certain qualitative percepts of speech (e.g., “breathiness”) play a role in diagnosis of speech disorders. A few studies have attempted to correlate some acoustic measures of HFE to perceptual ratings of disordered voice, with mild success. In Hammarberg et al. (1980), 14 voice clinicians rated short-story recordings of 17 patients with various voice disorders. Spectral levels of the frequency bands 0–2, 2–5, and 5–8 kHz were then compared in the LTAS of each recording. A significant correlation was found between the rating of “breathy” and the slope of the LTAS. Specifically, breathy voices were characterized by a steep decrease in spectral level from the 0–2 kHz band to the 2–5 kHz band, while the 5–8 kHz band had nearly the same level as the 2–5 kHz band. Conversely, “vocal fry/creaky” voice ratings were correlated with a steep decrease in level from the 2–5 kHz band to the 5–8 kHz band. Later, de Krom (1995) reported a negative correlation between perceived breathiness and the difference in the level of these two higher bands (subtracting the 5–8 kHz band level from the 2–5 kHz band level), though this measure explained little variance in “breathiness” and “roughness” ratings. de Krom (1995) also examined harmonics-to-noise ratio in this highest band, but again found it to explain little variance.

More recently, Liss et al. (2010) attempted to classify four different groups of speakers with ataxia, amyotrophic lateral sclerosis, Huntington’s disease, Parkinson’s disease, and one group of normal speakers using several speech rhythm metrics extracted from the amplitude envelopes of separate octave bands of the speech signal. One metric extracted from the 8-kHz octave was found to be most predictive of group classification, suggesting that attending to HFE could be an effective strategy in the diagnosis of speech disorders.

### LOCALIZATION

As discussed at the beginning of this review, Rayleigh’s (1907, 1908) original reports on the phenomenon of front/back errors in the localization of speech attributed it to the missing “high elements of the sound.” These reports followed up phenomena he observed some 30 years earlier that subjects could distinguish the front/back direction of a human voice source, but not of a simple sinusoid source (whistle, tuning fork): “The possibility of distinguishing a voice in front from a voice behind would thus appear to depend on the compound character of the sound in a way that is not easy to understand…” (Rayleigh, 1876). It has now been verified by Best et al. (2005) that listeners’ ability to localize speech stimuli is dependent upon HFE. When five listeners were asked to identify the direction of a speech source presented over headphones in virtual auditory space, low-pass filtering speech stimuli at 8 kHz led to a significant increase in errors in elevation in the median plane. In contrast, no significant change was found in errors in azimuth. By decreasing the level of energy above 8 kHz in a stepwise fashion (20-dB steps), systematic and significant decreases were seen in the accuracy of speech localization in elevation. Their results accord with localization research using non-speech broadband stimuli (Bronkhorst, 1995; Langendijk and Bronkhorst, 2002).

### TALKER RECOGNITION

There is some evidence that HFE contains features specific to an individual talker and is, therefore, useful in identifying different talkers. Schwartz (1968) presented 10 normal-hearing young adults with a gender discrimination task consisting of 1-sec recordings of isolated fricative productions of /f/, /θ/, /s/, and /ʃ/ produced by 9 males and 9 females. The overall accuracy achieved for each fricative was 93% for /s/, 90% for /ʃ/, 74% for /f/, and 69% for /θ/ (however, the latter two percentages were not significantly above chance). In an attempt to explain these results, he presented average spectra for /s/ and /ʃ/ showing gender differences in peak location of these fricatives (some of which were in the HFE region), while predicting that the broadband nature of /f/ and /θ/ would make gender discrimination more difficult. (These results are consistent with the spectral differences found later by Jongman et al., 2000).

White (2001) also performed a gender discrimination study, but used singing recordings from children (ages 3–12 years). Five teachers of singing for children were asked to identify the gender of the child, and to rate their own confidence in their decision on a 7-point scale. Two groups were examined: (1) boys most often correctly and confidently identified; and (2) girls most often correctly and confidently identified. Comparison of the mean spectra for each group revealed that the most obvious differences were above 4 kHz, including a broad peak centered at 5 kHz in the spectrum of singing produced by young boys. White also showed that the mean spectra of those girls who were *incorrectly* but confidently identified (i.e., identified as boys) exhibited the same spectral characteristics in the high-frequency region as for the correctly identified boys, and vice versa for the incorrectly identified girls. She suggested that listeners may have used the 5-kHz peak as a cue to distinguish young boys’ singing from that of young girls.

Hayakawa and Itakura (1994) have shown that an automated speaker recognition system achieved better recognition rates (of 15 male Japanese speakers) when HFE was included in the feature extraction and analysis. Rates steadily increased as the bandwidth was increased from about 88% at 6 kHz to about 96% at 16 kHz. They later showed that introducing white noise in the speech signal from 4 to 10 kHz did not have as severe an effect on feature extraction and automated speaker recognition performance as introducing noise from 0 to 4 kHz (Hayakawa and Itakura, 1995).

### INTELLIGIBILITY

Somewhat surprisingly, HFE affects the intelligibility of speech. While Rayleigh (1908) initially reported on the difficulty in distinguishing /s/ from /f/ without use of the high frequencies (see also Campbell, 1910), later studies on the articulation index seemed to indicate that HFE did not have a significant effect on intelligibility. However, a perceptual study performed by Lippmann (1996) showed that consonant identification was maintained for a degraded speech signal with the aid of HFE. He notch-filtered (combined low-pass and high-pass filtering) one female talker’s CVC speech samples using a low-pass frequency of 800 Hz and high-pass frequencies of 3.15, 4, 5, 6.3, 8, and 10 kHz. This filtering, in effect, removed nearly all of the frequency components traditionally recognized as containing the necessary information for speech intelligibility. Sixteen different consonants were represented in the CVC lists recorded. He presented these samples to three normal-hearing listeners monaurally, and found that consonant identification gradually decreased from 92% correct at 3.15 kHz to 75% correct at 8 kHz. At 10 kHz, scores dropped to 53% correct. Scores fell to 44% correct when only the low-pass filtered portion (800-Hz cutoff) was presented.

The sharp decrease in accuracy from the 8-kHz condition to the 10-kHz condition suggests that this 2-kHz band carries useful information for consonant identification, at least for female speech. Importantly, these percentages severely contradict predictions of intelligibility based on the articulation index. Lippmann concluded that the frequency region above 8 kHz contains sufficient (though perhaps redundant) information for consonant identification and discrimination. His stimuli included only female speech, however, and Stelmachowicz et al. (2001) presented evidence that perception of HFE is more important for female and child speech than for male speech.

While Lippmann (1996) did not address whether intelligibility information in HFE is useful in natural full-bandwidth speech, Apoux and Bacon (2004) later demonstrated that listeners (*n* = 6) gave the highest weighting to a band including HFE (3.5–10 kHz in their study) for consonant identification of spectrally degraded CV syllables (using 21 different consonants) and VC syllables (22 consonants) in noise. Using both male and female speech, their results showed that normal-hearing listeners’ identification scores for consonants in noise were significantly lower with the removal of this frequency band than with removal of one of the lower frequency bands (0–1.12, 1.12–2.25, or 2.25–3.5 kHz). Work by LeGendre et al. (2009) has suggested this may be related to the temporal structure and rhythm of speech. In analyzing signal amplitude modulation in separate octave bands of dysarthric speech, they found, remarkably, that the amplitude modulation of the 8-kHz octave band was most predictive of overall intelligibility. They suggested that listeners used this band to help segment syllables and words. [Note also that if competing noise in the environment drops off in level at higher frequencies more than does the speech spectrum, giving higher weighting to HFE for intelligibility of speech in noise would be an effective strategy. Furthermore, talkers speaking in noisy environments could make modifications that enhance HFE cues (Monson et al., 2012a)].

Perhaps the most compelling piece of evidence of the importance of HFE is a finding by Pittman (2008) regarding child word-learning ability. In this study hearing-impaired and normal-hearing children were exposed to five nonsense words spoken by a female talker (/saθnɘd/, /daztɘl/, /fasnɘʃ/, /stamɘn/, and /hamtɘl/), and their word-learning rate was monitored. The children were placed into two listening groups wherein they either received all nonsense words low-pass filtered at 4 kHz or low-pass filtered at 9 kHz. For both populations (hearing impaired and normal-hearing), the 4-kHz cutoff group required three times as many exposures as the 9-kHz cutoff group to learn the new words. This study built upon and corroborated the previous similar work of Stelmachowicz et al. (2001, 2007).

One research group has conducted a few studies showing that HFE has some effect on intelligibility. First, Moore et al. (2010) reported a small but significant benefit in intelligibility for normal-hearing listeners by increasing the cutoff frequency of low-pass filtered male speech from 5 to 7.5 kHz when target speech and background noise (two male talkers) were spatially separated. This group also presented male and female speech stimuli band-pass filtered at 5–10 kHz to normal-hearing listeners, reporting that listeners claimed they could recognize a little over 50% of the words (Füllgrabe et al., 2010).

Badri et al. (2011) studied differences between normal-hearing listeners and listeners who self-reported speech recognition problems in noise despite having clinically normal audiograms. While no significant differences were found in normal audiometric thresholds (up to 8 kHz) between the two groups, the impaired group—who did indeed perform worse on a sentence-in-noise perception test—showed worse thresholds at frequencies of 10, 12.5, and 14 kHz, though differences only reached significance for the latter two frequencies.

Finally, Berlin (1982) reported several case studies of individuals who had poor hearing at most conventional audiometric frequencies, but relatively good hearing in the HFE range. The first case he discovered, a 22-year-old woman, had pure-tone thresholds greater than 70 dB HL for audiometric frequencies up to 8 kHz. She was described by Berlin as having “a high-pitched, hyponasal, high-tone voice yet virtually perfect articulation, especially of fricatives” and “excellent language expression and comprehension and remarkably precise articulation.” He discovered that she exhibited much better pure-tone thresholds at frequencies above 8 kHz, and concluded that her communication ability must be attributed to this region of her hearing (what he termed “ultra-audiometric” hearing). Moreover, Berlin (1982) presented results of a study wherein certified audiologists, speech pathologists, and teachers of the deaf were asked to subjectively rate the quality of the speech of 104 individuals who had hearing loss of varying type and severity, using a school grade scale (*A–F*). He reported, “…every patient who received an *A* for speech (*n* = 6) had considerable residual ultra-audiometric hearing and, conversely, every patient who received an *F* for speech (*n* = 28) had measurable hearing only in the frequencies below 3,000 Hz.”

**Table 2** summarizes the major findings reported here regarding the perceptual importance of HFE.

**Table 2:** Summary of evidence for the perceptual importance of HFE.

Quality	
Listeners use sound quality terms to describe changes in vocal energy produced at frequencies above 5 kHz	Olson (1947), Monson et al. (2011, 2014)
Relative spectral level of vocal energy at frequencies above 5 kHz correlates with ratings of breathiness	Hammarberg et al. (1980)
Speech naturalness scores are affected dramatically by frequencies between 7 and 10.9 kHz	Moore et al. (2008)
Listeners prefer the pleasantness and clarity of speech low-pass filtered at 10 kHz to speech low-pass filtered at 7.5 kHz	Füllgrabe et al. (2010)
**Localization**
Front-back errors increase significantly and systematically for speech low-pass filtered at 8 kHz	Rayleigh (1876, 1907, 1908), Best et al. (2005)
**Intelligibility**	
Hearing-impaired individuals with residual hearing above 8 kHz exhibit well-articulated speech	Berlin (1982)
HFE assists in consonant recognition when low-frequency spectral energy is degraded	Lippmann (1996), Apoux and Bacon (2004)
Children require three times as much exposure to learn novel words when deprived of speech energy at frequencies above 4 kHz	Pittman (2008)
Speech energy at frequencies above 5 kHz provide a significant benefit for intelligibility when target speech and background noise are spatially separated	Moore et al. (2010)
Poor audiometric thresholds above 8 kHz are associated with poorer speech-in-noise performance	Badri et al. (2011)

## CONCLUSION

The results from these studies addressing the acoustical and perceptual significance of HFE indicate that, while comprising a small amount of energy in the speech and voice spectrum, HFE affects at least percepts of quality, localization, and intelligibility (**Table 2**). There is compelling evidence that suggests that children use HFE during language development (Pittman, 2008), that HFE in isolation aids in learning speech (Berlin, 1982), that quality suffers dramatically when HFE is removed (Moore and Tan, 2003), and that adult listeners detect HFE level differences in everyday speech (Monson et al., 2011, 2014). These results indicate that we are in need of better understanding of the acoustic cues provided by HFE in speech. Thus in-depth study of speech HFE perception is merited.

The potential benefits of this research are germane to a variety of research areas within the speech, language, and auditory sciences. The possibility that there is substantial relevant and accessible linguistic information in HFE has implications for the development of cochlear implants, hearing aids, cell phones and other electronic communication technologies that are just now beginning to transmit this frequency range (Moore, 2012; Pulakka et al., 2012). Research on HFE should elucidate the best way to represent HFE cues to hearing-impaired listeners currently deprived of HFE. Speech recognition models incorporating HFE may turn out to be more robust and efficient than current models. Speech synthesis and vocal tract modeling techniques including HFE could be developed for more natural sounding speech and voice. Voice therapies and teaching focused on voice quality could be improved by an increased understanding of the role of HFE in voice quality. It is also possible that HFE plays a role in normal and abnormal language development and could be incorporated into models and therapies for communication impairments.

The studies to date generate several questions regarding HFE. From a production standpoint, what are the causes of spectral differences in HFE between subjects? What are the generation mechanisms of HFE and can HFE be produced and/or modified by learned behaviors? Since HFE affects perceived quality, is there a spectral shape for HFE that is optimal for speech or singing and how is this attained? From a perceptual standpoint, what other percepts are affected by HFE? What modifications to HFE will cause a shift in perception? How robust is the perceptual information in HFE? Why do some listeners show greater sensitivity to HFE changes than others? Does HFE indeed play a more important perceptual role during childhood development than during adulthood? The potential benefits and the prospect of developing more complete theories of speech and song production/perception provide motivation for expanded research into this upper end of the speech spectrum. We propose that future research efforts from the communication sciences be made in this direction.

## Conflict of Interest Statement

The authors declare that the research was conducted in the absence of any commercial or financial relationships that could be construed as a potential conflict of interest.
